# Healing through balance: Empowering benign paroxysmal positional vertigo individuals with nurse-directed interventions

**DOI:** 10.6026/973206300211969

**Published:** 2025-07-31

**Authors:** Vasanth Pandian Thommai Antony Savari Muthu, Suresh Kumar N., Shankar Shanmugam Rajendran, Duraikannu Anandhi, Padmavathy Murugan, Pradeepa Govindharaj, Athya Farheen Mohammed Sulthan

**Affiliations:** 1 Department of Medical Surgical Nursing, College of Nursing, Madras Medical College, Chennai, Tamil Nadu, India; 2 Institute of Otorhino Laryngology, MMC & RGGGH, Chennai, Tamil Nadu, India; 3 Department of Child Health Nursing, College of Nursing, Madras Medical College, Chennai, Tamil Nadu, India

**Keywords:** Benign paroxysmal positional vertigo, functional balance, physical performance, nurse-directed interventions, fall prevention

## Abstract

The effect of nurse-directed interventions, including the Epley procedure and fall-prevention education, on physical performance and functional balance in patients with Benign Paroxysmal Positional Vertigo (BPPV) is of interest. 50 participants were divided into an experimental group (Epley technique and mobility education) and a control group (standard care). After the intervention, the experimental group showed significant improvements in the Short Physical Performance Battery (SPPB) and Berg Balance Scale (BBS) scores, with a positive correlation (r = 0.38, p = 0.01) between physical performance and balance. Socio-demographic factors also influenced outcomes. Nurse-directed interventions were found to be effective, low-cost methods for improving function and reducing fall risk in BPPV patients.

## Background:

Benign Paroxysmal Positional Vertigo (BPPV) is a common vestibular disorder affecting 2.4% of the global population, especially older adults and women [1, 2]. It causes sudden vertigo episodes triggered by specific head movements, due to dislodged otoconia in the semicircular canals, disrupting balance signals to the brain [3]. This results in dizziness, nausea and balance issues, severely impacting physical function. Around 60% to 80% of BPPV patients struggle with tasks requiring head movement [4] and 35% to 50% experience falls or near-falls annually [5], leading to restricted activity, muscle deconditioning and reduced mobility [6]. Additionally, 70% of patients face challenges with balance-related activities, increasing social isolation, anxiety and depression [7, 8]. The Epley manoeuvre, a key treatment, is effective in 60% to 92% of cases, providing immediate relief by repositioning otoconia [9]. Educating patients on safe movements can reduce vertigo and falls by about 30% [10]. Therefore, it is of interest to describe the impact of nurse-directed therapies on physical performance and functional balance in BPPV patients, emphasizing the role of nurses in improving patient outcomes and quality of life.

## Materials and Methods:

A quasi-experimental, non-randomized control group design was used to evaluate the efficacy of nurse-directed therapies on physical performance and functional balance in patients with BPPV. The research was carried out at the Rajiv Gandhi Government General Hospital in Chennai after the approval from Institutional Ethics Committee, Madras Medical College and Chennai (IEC-MMC/Approval/58112024). A total of 50 patients diagnosed with BPPV were recruited by a non-probability convenience sample procedure and evenly divided into two groups: 25 participants in the experimental group and 25 in the control group. Individuals aged 40 years and older, diagnosed with BPPV and capable of adhering to instructions were included in the study. Patients with central nervous system illnesses, recent cranial injuries, or significant cognitive deficits were excluded from the study. Baseline physical performance and functional balance levels were assessed in both groups using the Short Physical Performance Battery and the Berg Balance Scale. The experimental group underwent nurse-directed interventions, which included the Epley maneuver and a teaching booklet on safe movement practices and fall-prevention strategies. The interventions were executed over a four-week period. The control group received conventional medical treatment without any additional intervention. Post-intervention measures, utilizing the SPPB and BBS, were performed for both groups at 28th day. The study was initiated following approval by the Institutional Ethics Committee, Madras Medical College, Chennai and formal written consent from the Director of the Institute of ENT, RGGGH and Chennai. Written informed consent was obtained from all participants. Pre-tests, including sociodemographic and clinical questionnaires, the Short Physical Performance Battery and the Berg Balance Scale, were administered, taking 10-15 minutes per participant. The participants were then divided into experimental and control groups. The experimental group received a 30-40 minute nurse-directed intervention comprising an educational booklet on safe movement and fall prevention techniques, along with the Epley maneuver, while the control group received routine teaching. After 28 days, a post-test was conducted. Data were coded in Microsoft Excel and analyzed using IBM SPSS Version 26 to assess the effectiveness of the intervention. Demographic variables were presented as frequencies and percentages, while performance scores were reported as means and standard deviations. Chi-square tests were used to assess group similarities and score relationships Independent and paired t-tests analysed between the groups. p-value ≤ 0.05 indicated statistical significance.

## Results:

The mean age of the participants was 37.5 ± 10.0 years. In both groups, males constituted the majority, comprising 80% of the experimental group and 72% of the control group. Married individuals constituted the majority, representing 88% and 80%, respectively. Urban residence constituted 52% of the experimental group and 56% of the control group. In terms of education, 36% of the experimental group and 44% of the control group possessed a degree or higher. Private sector employment was recorded at 60% in the experimental group and 68% in the control group. The majority of individuals adhered to a varied diet. Caffeine intake was seen in 44% of the experimental group and 68% of the control group. The majority of individuals experienced benign paroxysmal positional vertigo for 1 to 2 years, with 40% in each group. Family history was predominantly absent, spinal abnormalities were absent and functional independence was prevalent across all groups, with no notable differences. No substantial differences were detected. During the pre-intervention evaluation, mild physical performance restrictions were prevalent, affecting 68% of the experimental group and 60% of the control group; the remaining 32% and 40%, respectively, reported only negligible impairment. Both group had moderate or severe limits and the inter-group variance was statistically insignificant (χ^2^, p > 0.05). Regarding fall risk, a moderate risk was observed in 80% of the experimental group, compared to 72% of the control group. Low risk was present in 20% and 28%, respectively, with no participants classified as high-risk. Once again, the differences were not statistically significant. Following the intervention, 80% of the experimental group exhibited minor limitations, while 20% showed mild restrictions. In contrast, the control group demonstrated 52% with minimal impairment and 48% with mild impairment. The χ^2^ test validated a significant intergroup difference, indicating improvement following the nurse-directed program. In the fall risk assessment, the proportion of low-risk cases increased to 76% in the experimental group, compared to 36% in the control group. Meanwhile, the proportion of intermediate-risk cases decreased to 24% versus 64%, with no high-risk cases, resulting in significant differentiation. Following nurse-directed interventions, the experimental group exhibited improvements in physical performance and balance. Their mean SPPB score increased from 9.04 at baseline to 11.48 at follow-up, representing a 20.33% improvement, whereas the control group experienced a 2.33% increase from 9.24 to 9.52.

The functional balance demonstrated that in the experimental group, the mean increased from 34.88 to 47.88, a 23.21% gain, whereas the control group progressed from 36.00 to 38.32, a modest 4.14% improvement. These data demonstrate that nurse-directed interventions improve the physical performance and functional balance in patients with BPPV (Table 1 - see PDF). Age and family type showed significant correlations with physical performance (χ^2^ = 9.47 and χ^2^ = 6.75, respectively; p < 0.05). Individuals aged 46-55 and those from joint households exhibited greater mild restrictions. Autonomous people exhibited fewer restrictions (88.89%) compared to those with partial dependence (57.14%), indicating a significant correlation (χ^2^ = 4.26; p < 0.05). Age, domicile and family type were significant factors for fall risk (χ^2^ = 8.92, χ^2^ = 6.18 and χ^2^ = 5.72, respectively; p < 0.05), with younger, urban and nuclear-family participants exhibiting a higher moderate risk. Independence was associated with a reduced incidence of falls (χ^2^ = 5.32; p < 0.05). Other variables exhibited no significant correlation. In the experimental group, the mean functional balance score increased significantly from 34.88 (62.29%) in the pretest to 47.88 (85.50%) in the posttest, showing a 23.21% improvement. In contrast, the control group showed only a minimal increase, with the mean score rising from 36.00 (64.29%) to 38.32 (68.43%), indicating a 4.14% improvement. These results demonstrate that nurse-led interventions had a substantial positive impact on enhancing balance in BPPV patients compared to standard care (Table 2 - see PDF).

## Discussion:

This study confirms that patients with benign paroxysmal positional vertigo frequently present with mild physical performance limitations and a moderate fall risk, similar to observations made by Huang *et al.* (2025) and Pauwels *et al.* (2024) [11, 4]. Nurse-directed vestibular interventions produced clinically meaningful gains: the experimental group's Short Physical Performance Battery rose by 20.33 % and functional-balance scores by 23.21 %, whereas controls improved only 2.33 % and 4.14 %, respectively. These outcomes parallel the rapid symptom resolution reported by Chen *et al.* (2023) [12] using the modified Epley manoeuvre and the balance benefits of combined vestibular exercise programs noted by Taçalan *et al.* (2021) [13], underscoring the pivotal role of nurses in translating evidence-based manoeuvres into routine care [12, 13]. Chang *et al.* [14] specifically highlighted that even in cases of peripheral vestibular dysfunction, functional impairments can significantly influence patients' balance and mobility, reinforcing the need for early identification and targeted intervention. Routine functional screening and early referral for vestibular rehabilitation should therefore be embedded in BPPV management pathways, particularly for middle-aged adults in joint households and urban settings. Limitations include the single-centre design, modest sample size and short follow-up; future multicentre trials with longer monitoring are warranted to validate durability and generalisability of nurse-directed interventions.

## Conclusion:

Nurses play a critical role in improving physical performance and balance in BPPV patients through patient-centered therapies such as the Epley manoeuvre and fall-prevention guidance. Their combined approach of manual treatment, education, and emotional support enables effective symptom management and restoration of daily functioning. Ongoing assessment and individualized care position nursing professionals as key contributors to successful vestibular rehabilitation and enhanced quality of life.

## References

[1] Zhou F (2022). Front Neurol..

[2] Kim HJ (2023). JAMA Neurol..

[3] Madrigal J (2024). Cureus..

[4] Pauwels S (2023). J Neurol Phys Ther..

[5] Donovan J (2023). Clin Rehabil..

[6] Bazoni JA (2014). Int Arch Otorhinolaryngol..

[7] Hu Y (2023). Front Neurol..

[8] Özgirgin ON (2024). Front Neurol..

[9] Alfarghal M (2023). Front Neurol..

[10] Yetiser S (2022). Clin Med Res..

[11] Huang X (2025). Aging Clin Exp Res..

[12] Chen X (2023). Front Neurol..

[13] Taçalan E (2021). J Bodyw Mov Ther..

[14] Chang WC (2006). Otolaryngol Head Neck Surg..

